# Metastatic Lesion of the Tibia from Renal Cell Carcinoma

**DOI:** 10.1155/2021/2428820

**Published:** 2021-07-30

**Authors:** Piotr Młodożeniec, Krzysztof Balawender, Mateusz Zasadny

**Affiliations:** ^1^Clinical Department of Urology and Urological Oncology, Municipal Hospital in Rzeszow, Poland; ^2^Morphological Sciences Department, Institute of Medical Sciences, Medical College of Rzeszow University, Poland

## Abstract

**Introduction:**

Renal cell carcinoma is responsible for 3% of all cancers, with the highest incidence occurring in Western countries. Additionally, in patients with osseous metastasis, only 3% occur within the tibia. Rarely, a patient presents with a primary complaint of lower limb pain in advanced metastatic renal cell carcinoma. *Case Presentation*. The patient arrived at the emergency department with a primary complaint of left ankle pain. Ankle X-rays demonstrated a lytic lesion involving the medial malleolus with possible metastatic disease. CT scan confirmed a tumor within the right kidney. The patient was treated with a laparoscopic radical nephrectomy with histopathologic confirmation of clear cell renal cell carcinoma. Biopsy was then performed of the tibial lesion, confirming metastatic clear cell renal cell carcinoma. The tibial lesion was treated with local radiotherapy, and because of the progression of the tibia lesion, a decision was made to amputate the leg. Additionally, the patient was enrolled to sunitinib treatment and was disease free at one year of follow-up. 13 months after diagnosis of cancer, she was suffering a major stroke of the brain that caused her to die.

**Conclusion:**

The treatment of patients with osseous metastases of renal cell cancer depends on the number of metastases, location of metastases, and overall health of the patient. We performed an overview of available literature and provided a summary regarding the use of cytoreductive nephrectomy, local therapy, target therapy, and bone-targeting agents in the treatment of metastatic renal cell cancer.

## 1. Introduction

The number of patients diagnosed with oncologic disease has been on the rise. Of all cancers diagnosed, renal cell carcinoma (RCC) makes up 3% of all cases, with the highest incidence occurring in Western countries. In 2018, the European Union recorded 99,200 new cases of RCC and 39,100 kidney cancer-related deaths [1]. Metastatic disease in RCC is not uncommon. The most common sites of RCC metastases are lung (45.2–51.2%), bone (29.5–33.5%) lymph nodes (21.8–41.5%), liver (17–20.3%), adrenal (8.9%), and brain (8.1–9.8%) [2, 3]. In multivariable analyses, young age was an independent predictor for the presence of multiple concomitant metastatic sites (odds ratio: 1.2, *p* < 0.001) [2]. In only 10–20% of patients, bone metastases are found without additional metastatic involvement [2–4]. Most commonly osseous metastases occur in the spinal column (59%), sacrum (39%), and long bones (31%) [4]. At the time of diagnosis of RCC, 31% of patients had a bone metastasis present and 69% developed bone metastasis after RCC diagnosis. Of all patients with RCC bone metastases, 71% had multiple lesions and 29% had a single one [5].

To our knowledge, we report the case of a patient with advanced clear cell renal cell carcinoma (ccRCC) to present solely with manifestations of a bony metastasis. This work has been reported in line with the CARE criteria [6]. We believe this case further elaborates on the presentation of metastatic renal cell carcinoma and adds to the published literature on the topic.

## 2. Case Presentation

**Figure 1:**
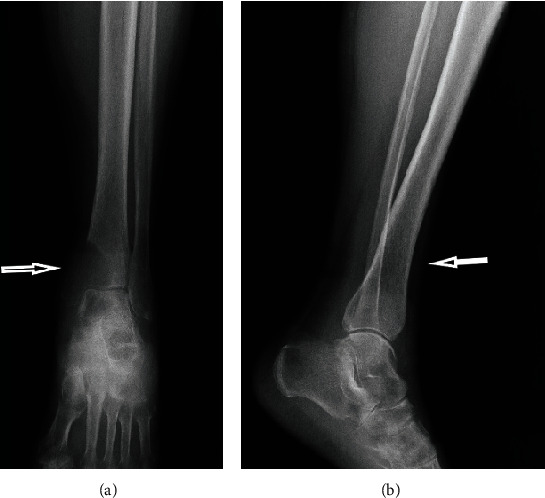
X-ray anteroposterior/lateral view of the leg showing destruction of the distal tibia.

**Figure 2:**
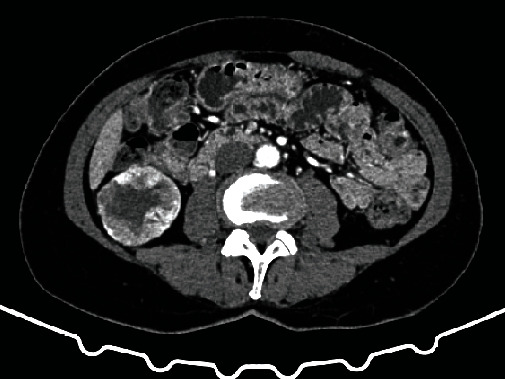
Computed tomography showed a solid mass measuring 40 × 36 × 30 mm in the right kidney.

**Figure 3:**
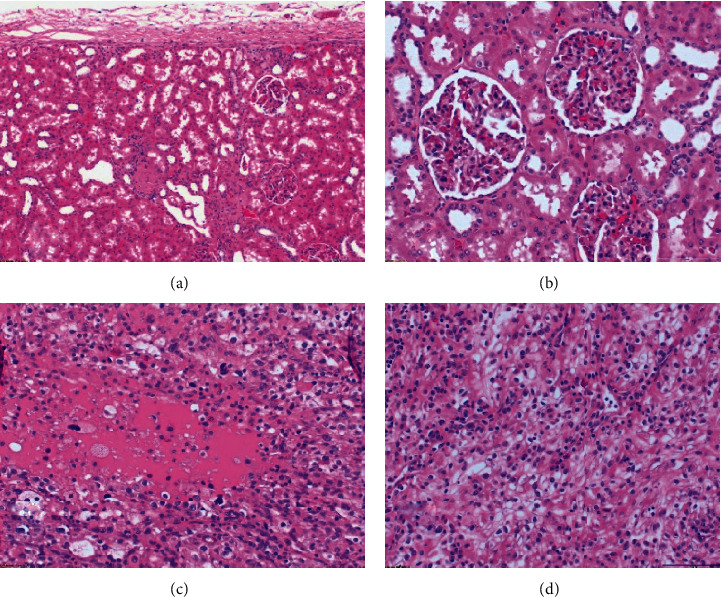
(a–d) The micrograph of a clear cell renal cell carcinoma H&E stain. This image demonstrates the optically clear tumor cells with uniform small nuclei without nucleolar ranged in an alveolar pattern. Cells with clear cytoplasm, typically arranged in nests. Nuclear atypia is common. Water-clear or optically clear cells (due to glycogen content) arranged in nests in “chicken wire” vasculatures. Visible subcapsular changes in the area of the kidney cortex. Numerous extravasations are visible in the vascular glomeruli (a, b). Metastasis of renal cell carcinoma. Histological microphotograph showing neoplastic cells with prominent nucleoli. Ankle joint and metastasis of renal cell carcinoma H&E sections (c, d). Scal bar: (a) 200 *μ*m; (b–d) 100 *μ*m. The samples were analyzed morphologically and photographed under an Olympus BX43 light microscope equipped with an Olympus SC50 digital camera.

A 59-year-old female presented to the emergency department in September 2019 with a chief complaint of left ankle pain and difficulty walking for 10 days. In physical examination, small swelling and the tenderness of the left medial ankle were found. A patient in an interview with no significant coexisting diseases and injuries accepts medicines for hypertension. Ankle X-rays (anterior-posterior and lateral views) were performed and demonstrated an osteolytic lesion within the medial malleolus and distal tibial metaphysis (Figure 1). Given the clinical picture, metastatic disease was suspected. Therefore, CT scans of the chest, abdomen, and pelvis were performed. A tumor of the right kidney was identified and presumed to be renal cell carcinoma (Figure 2). Surgical treatment of the RCC was undertaken with a laparoscopic radical nephrectomy. The procedure was completed without complication. The histopathologic report confirmed the diagnosis of ccRCC pT1b with angioinvasion with negative surgical margins, WHO/ISUP Grade 2 (Figure 3). Next, an aspiration biopsy of the tibial lesion was performed to confirm the diagnosis of metastatic ccRCC. The biopsy was completed without complication, and histologic diagnosis confirmed a metastasis of ccRCC (CD10+, vimentin+, TTFI–, RCC+, and PAX8+; Figure 3). Decision of immediate local radiotherapy on tibial lesion was taken. After 2 months due to progression of the tibial lesion, a decision was made to amputate the leg above ankle, upon counselling the patient. The patient underwent procedure. Postoperative recovery and rehabilitation were uneventful. Additionally, the patient was considered to be at intermediate risk according to the Memorial Sloan-Kettering Cancer Center (MSKCC) criteria and sunitinib treatment was started at 50 mg daily for two of every three weeks and was disease free at one year of follow-up. 13 months after diagnosis of RCC, she was suffering a major stroke of the brain that caused her to die.

## 3. Discussion

The predictors of cancer-specific mortality (CSM) in patients with ccRCC metastatic disease at the time of diagnosis are higher cT stage, higher histologic Fuhrman grade, or clinical nodal disease. Despite the fact that clear cell RCC is the most common type of metastatic RCC, sarcomatoid, papillary, and collecting duct type of RCC are associated with higher CSM [3]. Studies have shown the median survival time after diagnosis of skeletal metastasis in RCC to be 12 months [5], 15.8 months [7], and 32 months [8]. Furthermore, the number of bone metastases is inversely associated with overall survival (OS). Patients with 1, 2–5, or >5 bone metastases had a median overall survival of 28 months, 18 months, and 9 months, respectively [9]. The early diagnosis of bony metastases is important in reducing morbidity [10, 11], but formal recommendations do not advise the use of bone scan and/or positron-emission tomography CT in the staging of renal cell carcinoma. These modalities are limited to patients presenting with skeletal-related events (SRE) [12, 13]. The data about tibial metastases in RCC is scarce given the fact that tibial metastases occur in only 3% of all cases of renal cell carcinoma with bone metastases [7].

### 3.1. Treatment

The first issue of discussion in the treatment of RCC is if cytoreductive nephrectomy (CN) should be conducted. Chandrasekar et al. reported improved CSM with cytoreductive nephrectomy in a large population-based study [3]. Heng et al. further show improved OS with cytoreductive nephrectomy (HR: 0.60; 95% CI, 0.52–0.69; *p* < 0.001) [14]. Conversely, CARMENA, a phase III noninferiority RCT (randomized controlled trail) which found sunitinib alone, was not inferior in regard to OS when compared to CN and sunitinib therapy [15, 16]. European Association of Urology Guidelines 2021 recommends against cytoreductive nephrectomy in MSKCC poor-risk and intermediate-risk patients requiring systemic therapy with vascular endothelial growth factor receptor- (VEGFR-) tyrosine kinase inhibitor (TKI) but suggests cytoreductive nephrectomy in patients with good renal function who do not require systemic therapy. Additionally, CN is recommended in patients with oligometastases when complete local treatment of the metastases can be achieved.

### 3.2. Local Therapy

Treatment options for RCC metastases consist of surgery, radiotherapy, and ablation therapy. In the case of a solitary metastatic lesion, resection with tumor-free margins can improved 5-year overall survival significantly from 11% to 31% (*p* = 0.028) [7]. Alt et al. demonstrated that complete surgical resection of multiple RCC metastases was associated with a prolonged median cancer specific survival (CSS) to 4.8 years and, in fact, significantly improved the 5-year CSS rate (49.4%) compared with the rate among patients who did not undergo complete metastasectomy (13.9%) [17]. This benefit of metastatic resection has been confirmed by many other authors [18–20]. Therefore, wide resection with curative intent seems to be the primary approach to solitary or oligometastatic bone metastasis [13]. Patients who are unable to undergo metastasectomy can undergo radiotherapy as an alternative option to help control pain, preserve bone mineralization, and suppress spinal cord compression [21]. Svedman et al. have established in a phase II trial that using SRE for patients with primary and metastatic RCC provides a high degree of local control rate (98%) [22]. High single-fraction (24Gy) stereotactic body radiotherapy has the highest 3-year local relapse-free survival (LRFS) (88%) compered to low single-fractioned (<24Gy) stereotactic body radiotherapy (22%) and hypofractionated radiotherapy (17%) [23]. Radiofrequency ablation (RFA) can be utilized in the palliative treatment of patients with bone metastases for pain relief with a clinical efficiency of 69.7% [24]. Cryoablation is associated with a control-rate per lesion of 82% when used in the setting of bone metastases [25]. Zugaro et al. have shown similar pain relief (64% and 68%) and statistically significant increases in the quality of life (*p* = 0.001) using either technique, radiofrequency ablation, or cryoablation [26]. Chemotherapy however has been shown to be ineffective in the treatment of RCC [27]. Target therapy has become a new hope for patients with RCC metastatic disease. Tyrosine kinase inhibitors (TKIs) can slow the progression of existing bone lesions and reduce the formation of new bone lesions [28, 29]. The first-generation TKIs like sunitinib or sorafenib have improved overall survival (24 months versus 18 months; *p* < 0.01) [30]. Compared with sorafenib, sunitinib significantly decreased formation (*p* = 0.034) and prolonged time to occurrence of new bone lesions (*p* = 0.047) [28]. In the METEOR study, Choueiri et al. reported that treatment with cabozantinib improved median overall survival (21.4 vs. 16.5 months, *p* = 0.00026), progression-free survival (7.4 vs. 3.9 months, *p* < 0 · 0001), and objective response (17% vs. 3%, *p* < 0 · 0001) when compared to the mTOR inhibitor, everolimus [31]. In conclusion, cabozantinib should be used preferentially to treat bone metastases that are not amenable to local therapies [13]. Bisphosphonates (BT) are a group of bone targeting agents that work to decrease osteoclast activity. Before the era of target therapy, zoledronic acid decreased the rate of SREs compared to placebo (37% vs. 74%, *P* = 0.0015) [32]. The use of BT in conjunction with TKIs was not associated with improved OS (13.3 vs. 13.1 months, *p* = 0.3801), improved PFS (5.1 vs. 4.9 months *p* = 0.1785), and decreased rate of SREs compared with nonusers (8.6% vs. 5.8%, *p* = 0.191) [33]. During BT therapy, osteonecrosis of the jaw is possible, so patients should undergo regular dental examinations before and during treatment. Patients treated with zoledronic acid should undergo renal function monitoring [34]. The literature is inconclusive as to whether bone target therapy in addition to target therapy improves clinical outcomes. However, bone-targeting agents should be used to control tumor-associated hypercalcemia, with calcium and vitamin D supplementation [13].

## 4. Conclusion

Clear cell RCC is the most common type of renal cancer. Metastatic lesions of bone are the 3^rd^ most common site of all metastases with the leg being a rare site of RCC metastases. The median survival time after diagnoses of skeletal metastasis in RCC is 12–32 months. Early diagnosis of bone metastases is important for reducing morbidity. Cytoreductive nephrectomy is not indicated routinely, but only to patients with good renal function who do not require systemic therapy, or to patients with oligometastases when complete local treatment of the metastases can be achieved. Wide resection of metastases with curative intent seems to be the primary approach to solitary or oligometastatic bone metastasis. High single-fraction (24Gy) stereotactic body radiotherapy is the best option in palliative local treatment to patients who are not able to undergo metastasectomy. Chemotherapy is ineffective in the treatment of RCC. In systemic therapy, cabozantinib should be used preferentially to treat RCC with bone metastases. The beneficial effects of adding bone-target agents to TKI therapy in the setting of bone metastasis have not been seen, but in the setting of tumor-associated hypercalcemia, their use is recommended.
